# COVID-19 Impact on Healthcare and Supportive Services for People Who Use Drugs (PWUDs) in Malaysia

**DOI:** 10.3389/fpsyt.2021.630730

**Published:** 2021-03-29

**Authors:** Balasingam Vicknasingam, Nur Afiqah Mohd Salleh, Weng-Tink Chooi, Darshan Singh, Norzarina Mohd Zaharim, Adeeba Kamarulzaman, Marek C. Chawarski

**Affiliations:** ^1^Centre for Drug Research, Universiti Sains Malaysia, Penang, Malaysia; ^2^Department of Social and Preventive Medicine, Faculty of Medicine, Universiti Malaya, Kuala Lumpur, Malaysia; ^3^Centre of Excellence for Research in AIDS, University Malaya, Kuala Lumpur, Malaysia; ^4^School of Social Sciences, Universiti Sains Malaysia, Penang, Malaysia; ^5^Department of Medicine, Faculty of Medicine, University Malaya, Kuala Lumpur, Malaysia; ^6^Yale School of Medicine, New Haven, CT, United States

**Keywords:** people who use drugs, COVID-19, methadone, HIV, Malaysia

## Abstract

**Background:** Restrictive orders and temporary programmatic or *ad hoc* changes within healthcare and other supportive systems that were implemented in response to the COVID-19 epidemic in Malaysia may have created hindrances to accessing healthcare and/or receiving other supportive services for people who use drugs (PWUDs).

**Design:** A primarily qualitative study has been conducted to evaluate how service providers and recipients were adapting and coping during the initial periods of the COVID-19 response.

**Settings:** The study engaged several healthcare and non-governmental organizations (NGOs) in the peninsular states of Penang, Kelantan, Selangor, and Melaka.

**Participants:** Medical personnel of methadone maintenance treatment (MMT) programs (*n* = 2) and HIV clinics (*n* = 3), staff of NGO services (*n* = 4), and MMT patients (*n* = 9) were interviewed using a semi-structured format.

**Results:** Interviewed participants reported significant organizational, programmatic, and treatment protocols related changes implemented within the healthcare and support services in addition to nationally imposed Movement Control Orders (MCOs). Changes aimed to reduce patient flow and concentration at the on-site services locations, including less frequent in-person visits, increased use of telemedicine resources, and greater reliance on telecommunication methods to maintain contacts with patients and clients; changes in medication dispensing protocols, including increased take-home doses and relaxed rules for obtaining them, or delivery of medications to patients' homes or locations near their homes were reported by the majority of study participants. No significant rates of COVID-19 infections among PWUDs, including among those with HIV have been reported at the study sites.

**Conclusions:** Although the reported changes presented new challenges for both services providers and recipients and resulted in some degree of initial disruption, generally, all participants reported successful implementation and high levels of compliance with the newly introduced restrictions, regulations, and protocols, resulting in relatively low rates of treatment disruption or discontinuation at the study sites.

## Introduction

In response to the coronavirus (COVID-19) threat, Malaysia imposed several phases of the Movement Control Order (MCO) nationwide, beginning on March 18, 2020 (1). During the initial MCO phase, only essential services were allowed to operate; activities of educational institutions and religious services and organizations were suspended; restaurants, bars, entertainment outlets, cinemas were ordered to close; international and interstate travel was not permitted; locally, only those working for essential services were allowed to leave homes; all other citizens were asked to stay at home and only one person per each family living together was allowed to go out to obtain food, essential supplies, and medicine. Between March 18 and May 4, 2020, 5,563 COVID-19 cases in a population of 32.7 million people (2) were recorded in Malaysia. Subsequently, the Malaysian government imposed the Conditional Movement Control Order (CMCO) lasting from May 5 to June 10, 2020. During the CMCO period, most business and services were allowed to open, but entertainment outlets including cinemas, theme parks, religious and education institutions were ordered to remain closed. Interstate travel was allowed only for essential services, including food and medical transports. During the CMCO, 1,955 cases were recorded in the whole country. As the cases continued to decrease, the Recovery Movement Control Order (RMCO) was established between June 11 and August 31, 2020 and only 971 cases were recorded during this period (3). During this phase, more businesses were allowed to re-open. Large scale social, religious, education activities, and international and interstate travel were still not permitted. Throughout the MCO, CMCO, and RMCO all imposed restrictions were enforced by the law enforcement agencies and included police patrols in residential areas, road check-points, and by issuing citations and penalties for non-compliance.

Though these restrictive orders were intended to slow the spread of the COVID-19, and indeed they have shown considerable reduction of new infections in Malaysia, there is a concern that for people who use drugs (PWUDs), including those with substance use disorders (SUDs), the various types of measures to control or restrict peoples' movement, distancing or limiting social contacts, or restricting access to various social and healthcare facilities may have created particularly challenging hindrances to receiving social support or accessing healthcare and other supportive services (4–10).

The present study aimed to collect information and qualitative and quantitative data on the potential impact of the MCO on PWUDs in Malaysia to evaluate how service providers and recipients of these services were adapting and coping during this period in Malaysia.

## Methods

### Design

The study combined a qualitative component, consisting of interviews with key personnel, service providers, and SUD patients receiving treatment in participating clinics, and a quantitative component based on data from pre-MCO and during MCO/CMCO/RMCO periods from the MMT program at Sungai Buloh Hospital (SBH) in Selangor.

### Ethical Considerations

The study was reviewed and approved by the Human Ethics Committee of Universiti Sains Malaysia, Penang (protocol # USM/JEPeM/COVID19-30). A written informed consent was obtained from all study participants. No personally identifiable information has been collected from the interviewees and clinic records used in quantitative analyses were de-identified before accessing and analyzing.

### Locations and Timeline

The study was conducted in several locations in the peninsular states of Penang, Kelantan, Selangor, and Melaka. Selection of study sites was determined by the availability of healthcare and/or non-governmental organizations (NGO) facilities that could be engaged in the study research protocol.

Qualitative interviews collected information from healthcare and service providers, as well as patients with SUD receiving treatment or other services during the MCO/CMCO/RMCO periods from March 18 to August 31, 2020. Quantitative study component evaluated urine toxicology tests results collected before the MCO period (December 2019 to February 2020) and during the RMCO period (June 2020 to August 2020) at the MMT program at SBH in Selangor.

One infectious disease MD physician and two MD general practitioners from the HIV clinic and the methadone maintenance treatment (MMT) clinic, respectively, in SBH, Selangor; one MD physician from MMT clinic in Masjid Tanah, Melaka; and one nurse from Hospital Raja Perempuan Zainab in Kota Bharu, Kelantan were interviewed. A total of nine MMT patients were interviewed: four from Masjid Tanah, Melaka, and five from Kota Bharu. One programme manager and one programme coordinator at the AIDS Action Research Group (AARG) NGO in Penang; one programme coordinator from the Insaf Murni NGO in Selangor and one programme coordinator from the SAHABAT NGO in Kelantan were also interviewed. Data collection methods.

Qualitative interviews were based on a semi-structured interview guide developed for the current study. The interviews focused on the following domains of interest: (a) programmatic changes in healthcare policies and protocols implemented during MCO/CMCO/RMCO periods; (b) implemented operational changes at the point of care level and at supportive services facilities; (c) effects of the MCO/CMCO/RMCO restrictions and other implemented changes on provision of healthcare and supportive services; (d) effects of MCO/CMCO/RMCO restrictions and other implemented changes on patient access; and (e) effects of MCO/CMCO/RMCO restrictions and other implemented changes on substance use.

One participant was interviewed over the phone, all other interviews were conducted face-to-face. The interviewers wrote down answers to all questions and took additional notes as needed. Study personnel adhered to COVID-19 related regulations implemented by the Malaysian government pertaining to body temperature checks and being interviewed about potential symptoms and health status upon entering healthcare facilities, wearing face masks and maintaining social distancing during the interviews.

Deidentified urine toxicology test results for opiates, benzodiazepines, methamphetamine, amphetamine, and tetrahydrocannabinol collected routinely as part of clinical monitoring at the MMT clinic at SBH in Selangor between December 2019 and August 2020 were also obtained.

### Data Analytical Approaches

The analyses focussed on: (a) identifying information on changes in policies, protocols, operating procedures, and implemented practices, and (b) on evaluating potential impact of these changes and of COVID-19 related restrictions on healthcare access, and substance use among PWUDs during the MCO, CMCO, and RMCO in Malaysia.

Collated notes from all qualitative interviews were reviewed by the study research group to identify informational content (i.e., descriptions of changes in policies, protocols, operating procedures, and implemented practices) and analyzed thematically to identify common patterns pertaining to impact, adaptation, and coping both on organizational and individual levels.

Descriptive analyses were conducted using MMT clinic records data. The overall rates of urine toxicology test results positive for any illicit substances during each month of pre-MCO (December 2019 to February 2020) and RMCO (June 2020 to August 2020) were calculated, tabulated, and compared.

## Results

### Qualitative Interviews With Healthcare Workers

#### MMT Physicians

The interviewed physicians reported that between January 2020 and August 2020 there were 131 and 78 active patients, respectively, in Masjid Tanah, Melaka and SBH, Selangor MMT clinics. In both clinics, there were no reported COVID-19 infections among their MMT patients.

Prior to the MCO, take-home doses of methadone were provided according to the national guidelines to patients who were considered to be in a stable recovery, as determined by negative urine toxicology tests conducted randomly, at least once a month. Patients with continuous urine tests negative for all tested illicit substances (opioids, amphetamine, methamphetamine, benzodiazepines, THC) for at least 3 months were eligible for take- home methadone doses. The national guidelines for prescribing take home doses, allowed for eligible patients to initially receive 3 to 4 days of take-home doses, and subsequently the number of doses could have been increased for up to 2 weeks maximum, for patients who continued with a stable recovery (11). In both clinics, ~50 to 60% of the patients were receiving take-home doses before the MCO.

During the MCO, urine testing was suspended until June 2020 at the SBH MMT clinic, but not at Masjid Tanah clinic in Melaka. Methadone take-home dose regulations were relaxed in both clinics participating in this study. Almost all patients in both clinics received take-home doses. Those who previously did not receive take-home doses started receiving a 1-week supply of methadone daily doses, and those previously on weekly take-home regimen, were receiving a 2-week supply of take-home methadone doses. Take home doses for both clinics were dispensed in individual bottles for each day of dosing. Patients were instructed to consume one bottle each day and return the empty bottles when coming to the clinic for their next take-home doses supply. The clinic staff has not collected any self-report on medication adherence, due to the brevity of clinic visit during the MCO.

Only patients who were newly admitted to the MMT program during the MCO were required to come to the clinics daily during their initial dose titration period. However, there were very few new patients enrolling during the MCO. Only one new patient was reported in the Masjid Tanah, Melaka MMT clinic. No new patients were admitted during the MCO, CMCO, and RMCO at the MMT clinic at SBH.

Interviewed MMT personnel indicated that they would prefer to continue with the relaxed rules for the methadone take-home dosing to continue even after the COVID-19 restrictions are ultimately lifted. As of November 2020, The Masjid Tanah MMT clinic in Melaka continues to provide take-home methadone doses to the majority of their patients. The MMT clinic at SBH returned to the pre-MCO regulations regarding take-home dosing in July 2020.

Interviewed personnel reported that some patients missed their clinic visits and medication pick up visits during the MCO, CMCO, and RMCO, but beginning in July most of these patients reengaged with their clinics.

### Descriptive Data From MMT Clinic

Table 1 shows summaries of urine toxicology results for illicit substance use (opioids, amphetamine, methamphetamine, benzodiazepines, THC) among MMT patients in SBH. The rates of patients testing positive for any of the tested substances during the pre-MCO period (from December 2019 to February 2020) and during the post- MCO or during the RMCO months (from June to August 2020) did not differ substantially and ranged between 18 and 24% in both evaluated periods. In both evaluated periods benzodiazepines and methamphetamine and/or amphetamine were the most commonly detected substances.

**Table 1:** Urine toxicology tests results at the MMT Clinic at SBH during pre- and post-MCO periods.

**Pre-movement control order (MCO) months**	**Patients with urine tests positive for any illicit substance % (n/N)**	**Number of patients positive for tested substances**
December 2019	23 (17/74)	bzd (6), met (4), amp/mor (2), met/mor (2), met/amp/mor (1), THC (1), met/amp (1)
January 2020	23 (16/74)	bzd (5), met/amp (3), met (2), met/amp/mor (2), mor (1), THC (1), met/mor (1). met/THC/mor (1)
February 2020	18 (13/74)	met (5), bzd (4), met/amp/mor (1), bzd/mor (1), THC (1), met/amp (1)
**Movement Control Order (MCO) monthsMarch to May 2020**	No urine tests conducted	
Recovery Movement Control Order (RMCO) months	Patients with urine tests positive for any illicit substance % (n/N)	Number of patients positive for tested substances
June 2020	24 (18/74)	met (5), met/amp (3), bzd (3), met/amp/mor (2), met/amp/mor/bzd (1), mor (1), bzd/mor (1), THC (1), met/THC/mor/amp (1)
July 2020	19 (14/7)	bzd (4), met/amp (4), met (2), mor (1), met/amp/mor (1), met/THC (1), amp (1)
August 2020	23 (17/74)	bzd (5), met/amp (5), met/amp/mor (3), THC (2), met (1), met/THC/mor/amp (1)

*bzd, benzodiazepine; met, methamphetamine; amp, amphetamine; mor, morphine; THC, tetrahydrocannabinol*.

### HIV Clinics Personnel

Before the MCO has been declared, the SBH in Selangor had the largest HIV treatment programme in Malaysia, with a census of over 9,000 HIV patients. Coinciding with the declaration of the MCO the hospital has been designated as the primary treatment and coordinating center for COVID-19 patients. It has been reported that it had treated approximately 5,000 COVID-19 cases and there was a total of 12 COVID-19 related fatalities reported by the time of the study qualitative interviews. Among all HIV patients at the SBH, only one was reported to become infected with the COVID-19, received the same course of treatment as other COVID-19 patients, and subsequently fully recovered without any COVID-19 related sequalae.

There were significant programmatic, structural/facilities, and organizational changes implemented to decrease concentration/congestion of people on the hospital grounds and to follow newly implemented social distancing rules, as well as to accommodate the new role for the hospital and to facilitate care for the expected influx of COVID-19 patients. The HIV in-patient ward was converted into an inpatient COVID-19 treatment ward. Other wards were also converted or designated to treatment of COVID-19 patients as needed. Existing HIV in-patients were transferred to other wards within the hospital, with some patients transferred to different hospitals or facilities, while some of the HIV outpatients who were assessed to require more vigilant care were admitted as inpatients. The SBH stopped accepting new non-COVID-19 with the exception of any urgent walk-ins. All new cases were referred to other hospitals. The SBH began accepting new non-COVID-19 patients around early to mid-June, after interstate travel was permitted. Patients traveling from other states received letters to certify their travel for important health related reasons. Overall, the interviewed healthcare professionals stated that the greatest challenge in maintaining clinic services was fatigue and COVID-19 case overload due to staffing shortages.

During the MCO, HIV patients who were determined to be clinically stable had their previously scheduled on-site face-to-face medical evaluation appointments with the clinic personnel canceled or postponed by 1 month. Additionally, a telemedicine consultation service offered to clinically stable HIV patients receiving ART operating at the SBH since 2017 continued during the MCO, CMCO, and RMCO periods. This service, called *EZ Clinic*, aimed to ease patient flow through the on-site HIV clinic and to remove some of the challenges of healthcare access, by reducing delays in patient-provider contacts and reducing travel and time burden of an in-person visits for patients who could utilize the telemedicine service. Through the *EZ Clinic* healthcare providers were able to conduct a rudimentary patients evaluation, review laboratory test results, and provide a consultation for their patients.

It was reported that patients registered with the *EZ Clinic* were more likely to maintain regular contact with their treatment providers. On the other hand, patients who were not utilizing the *EZ Clinic* were more likely to miss their evaluation appointments during the MCO, CMCO, and RMCO periods.

For patients scheduled for an in-person visit, the HIV clinic nurse called the patients ahead of their appointment to evaluate their current health status before deciding if they need to come to the clinic. If the patients were clinically stable and generally doing well, they were asked not to come for their scheduled appointment. Patients attending their scheduled appointments in-person were not allowed to be accompanied by family members, which was very common before the MCO. All laboratory tests for stable patients were suspended during the MCO.

Prior to MCO, patients who were receiving Antiretroviral Therapy (ART) were required to come to the on-site pharmacy to receive a monthly supply of ART medications. During the MCO, ART patients were given three options: sending/mailing their medication to their home or to the healthcare facility that was nearest to their residence; or drive through pharmacy pickup service at the hospital; or a walk-in pick up of prepared medication supply at the hospital lobby. No medication shortages were reported during the MCO, CMCO, and RMCO periods.

### Staff of NGOs Services

Insaf Murni, an NGO that provides HIV-related services to key populations, operates in two towns within the Selangor state: Klang and Kajang. The AIDS Action Research Group (AARG) operates in Penang, and provides a broad range of services, including needle and syringe services and HIV testing and counseling at sites on the island and at the mainland. SAHABAT NGO operates in Kota Bharu, Kelantan and offers needle and syringe services, HIV testing and counseling and operates a home shelter for PWUDs.

The interviewed NGO staff reported that a day before MCO was implemented, outreach workers from Insaf Murni have distributed a three-week supply of needles and syringes at their community distribution locations frequented by the PWUDs. During the MCO, CMCO, and RMCO, their organizations temporarily stopped providing counseling, community HIV testing, and drop-in services to the clients. All NGOs reported that they increased their needle and syringe package for clients from 1 week to 2 or 3 weeks supply and added face masks and disinfectants/sanitisers to the packages. Clients came to the organization dispensing sites in the community to pick up their packages.

During the MCO period, new clients who were referred by existing clients were registered through phone calls, rather than through in-person visits. An increase in the number of PWUDs interested in being referred to MMT during the MCO has been reported. Insaf Murni NGO also reported an increase in request for HIV and Hepatitis testing among men who have sex with men (MSM) and from the transgender community during the MCO, CMCO, and RMCO periods.

During the MCO, the government begun offering financial assistance to people who lost their jobs and a 6-month property and vehicles loan moratorium was introduced. The NGOs started to help their clients to complete the necessary application documents and assisted them in the application process. One NGO have also reported to provide food to 70 transgender people by delivering the food packages to their homes and to 120 PWUDs by delivering the food to the health clinic.

Starting in May 2020, during CMCO, outreach workers at Insaf Murni restarted to transport clients to a health clinic for Hepatitis C treatment. Their outreach workers were provided with the sets of personal protective equipment (PPE) including a face mask, eye protection, isolation gown, and gloves for their off-site travel and community work. Outreach workers have used this opportunity to restart distribution of needles and syringes at locations frequented by PWUDs, within the 15 km radius of the two towns where this NGO has been operating. During RMCO, outreach workers continued engaging with PWUDs including referrals to MMT treatment and provision of 3-week supply of clean needles and syringes. Counseling sessions and HIV testing for PWUDs resumed in August 2020.

Collection of used needles and syringes, community HIV testing and counseling programs, and narcotic-anonymous meetings were suspended. Collection of used needles and syringes resumed at Insaf Murni during RMCO, but the rates of returned needles and syringes dropped from pre-MCO 75% to ~30%.

Some of the commonly reported challenges faced by the NGOs during the MCO, CMCO, and RMCO were difficulties reaching out to their clients, especially those who were living further away from the NGOs operating sites. To reach the clients living outside 10–15 km radius from the sites, the outreach workers needed to obtain a permission from the police. NGOs' case workers were also restricted in accompanying clients for their healthcare appointments. Some of the interviewed NGOs' staff remarked that during the initial stages of the MCO they were worried about potential shortages of needles, syringes, and other supplies due to the overall disruption in the supply chains in the country. However, no major shortages of such supplies were reported during the interviews.

### MMT Patients

Five of the nine interviewed MMT patients were also receiving ART, and three of these were additionally receiving Hepatitis C treatment during the time of their study participation. All five MMT patients on ART were residing in the SAHABAT NGO shelter home in Kota Bharu, Kelantan. Prior to MCO, residents of the shelter home received weekly counseling. However, this service was suspended when the MCO was declared, as there were local travel restrictions preventing counselors from traveling to the shelter. The on-site staff of the shelter home continued to help the patients to ensure daily ART and Hepatitis C medication adherence and took them for scheduled follow up visits with their HIV clinic treatment providers as their HIV clinic in Kelantan continued to provide in-person services for patients residing in the shelter home.

The interviewed patients confirmed that after the MCO has been implemented take-home doses were given to patients previously on a daily dosing regimen and those already receiving take-home methadone doses became eligible for up-to 2 weeks of methadone take-home dosing. One patient expressed a concern regarding his take-home doses. He said that he did not have a proper place to store the medication as he lived with two younger siblings and nephews and nieces. He was worried that they may accidentally consume his medication even though he kept it in a locked drawer.

Interviewed patients reported that their respective MMT clinics implemented body temperature checks while entering the clinics, and social distancing rules on the clinics' grounds. They were also required to register in the national COVID-19 contact tracing application. Overall, all patients reported that generally they have not had significant problems in getting their supply of medications and their treatment was not interrupted throughout the MCO period.

The interviewed patients expressed mixed views about availability of street drugs during the MCO. Some stated that the price of a packet of street heroin was unchanged while the quantity in each packet was somewhat reduced. Others, stated that the price of heroin increased during the MCO. They also expressed different opinions regarding availability of street drugs: some said that the supply/availability was reduced, while others reported no perceived changes in the supply or availability.

## Discussion

This primarily qualitative study evaluated whether the MCOs imposed in Malaysia in March of 2020 in response to the COVID-19 epidemic and the related changes in healthcare and social support services created particularly challenging hindrances for PWUDs. The study collected semi-structured interviews with medical personnel of healthcare services, staff of NGOs, and MMT patients in the peninsular states of Penang, Kelantan, Selangor, and Melaka.

While PWUDs, especially those who use opioids and amphetamine-type-stimulants (ATS), are vulnerable to respiratory and pulmonary health problems and they were feared to be at increased risk of infection and high rates of treatment discontinuation during the COVID-19 pandemic (12), no significant rates of COVID-19 infections among PWUDs, including among those with HIV have been reported at the study sites. Additionally, relatively low rates of treatment disruption or discontinuation during the initial periods of MCOs were reported by the personnel of sites engaged by the study.

Interviewed participants reported significant organizational, programmatic, and treatment protocols related changes implemented within the healthcare and support services in addition to nationally imposed MCOs. The main changes aimed to reduce patient flow and concentration at the on-site services locations, including postponing, or less frequent scheduling of in-person visits, especially for patients determined to be clinically stable. A greater utilization of telemedicine resources and greater reliance on telecommunication methods instead of in-person visits or contacts to maintain therapeutic or service engagements with patients and clients was also commonly reported. Both MMT programs and HIV clinics implemented significant changes in medication dispensing protocols, including relaxed rules for patients to obtain take-home doses, increases in the duration of take-home doses, and delivery of ART medications to patients' homes or locations near their homes. While these changes were meant to be temporary, in some study locations the modified/relaxed medication protocols were still in place after the study completion and may continue to be utilized in the future. In particular, despite the relaxation of the rules for eligibility of methadone take-home dosing, neither healthcare professionals nor patients reported significant challenges resulting from the expansion of methadone take-home regimens at the participating MMT clinics. Urine toxicology data obtained from the MMT clinic at SBH (see Table 1) indicates that there were no substantial increases in the rates of patients testing positive for illicit substances after the rules for methadone take-home dosing were relaxed. This data also illustrates that that there were no substantial changes in types of illicit substances used by MMT patients during the pre- and post-MCO periods.

Interviewed staff of NGOs reported challenges in accessing some clients, especially in locations further away from their organizations operation sites, primarily due to travel restrictions. They also reported temporary discontinuation of some of their services, including HIV testing and counseling, and any services necessitating face-to-face or close interaction with the clients. Other services, including needle and syringe distribution continued without major disruptions, due to procedural changes, adjustments, and adaptations. All needle and syringe programs reported providing increased number of needles and syringes in their distribution packets, and providing additional COVID-19 related supplies, such as face masks and disinfectants. Some NGOs also reported initiating additional services that were not typically offered during the pre-MCO period, for example, assistance with applications for new government assistance programs, or food distribution.

Overall, no major or only transient disruptions in provided healthcare and other supportive services were reported by the interviewed healthcare providers, NGOs' staff, as well as MMT patients. Based on the conducted interviews and evaluation of available clinic records, the present study has not obtained any evidence of substantially increased rates of treatment or service discontinuation. Some increases in services demands (e.g., increased number of MMT referral inquiries) were also reported. Interviewed participants reported challenges related to travel/movement restrictions, and concerns about potential adverse effects of the disruptions in the supply chains on availability of medications and service supplies, however the study participants have not reported medication shortages or other significant treatment or supporting services disruption.

## Limitations

Due to COVID-19 response burden on healthcare and other social services, as well as travel restrictions being still in place when the study was conducted, the study was able to engage only a limited number of services, and a relatively small number of healthcare providers, NGO staff, and patients were enrolled. Study findings are based primarily on qualitative interviews with only limited quantitative data obtained and analyzed. Consequently, the study findings represent only a snapshot picture. A broader range of changes and adaptations were likely being implemented in different locations throughout Malaysia in addition to the nationwide imposed MCOs.

Despite these limitations, the study provides an overview of successful changes and adaptations that were implemented in Malaysia in response to the COVID-19 pandemic and outlines their potential impact on provision and access to healthcare and other supportive services for PWUDs in Malaysia. The study findings may inform future responses to potential crises and hindrances concerning provision of healthcare and social support services for PWUDs in Malaysia and other countries in the region.

## Conclusions

The reported changes and adaptations introduced to cope with the COVID-19 pandemic in Malaysia presented new challenges for both service providers and recipients and resulted in some degree of initial disruption. However, generally, all participants reported successful implementation of the changed or newly implemented procedures or protocols and high levels of compliance with the newly introduced restrictions, regulations, and protocols. The reports collected during the study indicate that both the personnel and patients or clients receiving services at the evaluated services were able to adapt well to the changes, resulting in relatively low rates of treatment or service disruption or discontinuation at the study sites.

## Data Availability Statement

The data that support the findings of this study are available on request from the corresponding author.

## Ethics Statement

The studies involving human participants were reviewed and approved by Human Ethics Committee of Universiti Sains Malaysia. The patients/participants provided their written informed consent to participate in this study.

## Author Contributions

NAMS, W-TC, NMZ, and DS were involved in the data collection. BV and MCC conducted the data analysis and drafted the manuscript. All authors contributed to the design, conceptualization of the study, revised and edited the manuscript, contributed to the article, and approved the submitted version.

## Conflict of Interest

The authors declare that the research was conducted in the absence of any commercial or financial relationships that could be construed as a potential conflict of interest.
